# A Case in Obstetrics

**Published:** 1898-12

**Authors:** 


### A CASE IN OBSTETRICS.

Medical "experience meetings" sometimes result from the discussion of unusual cases reported at medical societies, and the fruit is generally prophylactic in confessions, warnings, and expostulations, which edify the doctor and make to the public good. But it must be rare, we take it, that a physician can be found so self-sacrificing that he would of his own election go before the world in print with such an experience as decorates the columns of the October number of our esteemed contemporary, *The Medical Progress.* We quote *verbatim ad literatim,* but withhold the name of the contributor and his local habitation:

On April 25, 1898, I was called to see Mrs. K., aged thirty-five years. I had to go a distance of twelve miles. The husband, who came after me, was not in a very great rush, as most men are on such occasions. I was at the bedside of patient in an hour after message reached me. On inquiry found the child had been born three and a half or four hours, the cord had been cut, and an attempt to deliver the afterbirth had been made. The old lady who acted as Ach said "the afterbirth was completely grown to the woman." On examination I found the mouth of womb slightly tense, but would yield on pressure; manipulation over fundus of womb would bring on pains and a slight hemorrhage would start at each pain, which made me uneasy, as I knew the danger of delivering afterbirths that have been retained as long as three or more hours.

I gave her ergot, witch hazel, and other remedies to check and prevent hemorrhage. I waited half an hour, then told the husband the chances were against his wife. The remedies made but little, if any, impression on contractility of womb, for blood would gush at every pain.

I explained the trouble that was likely to follow if I delivered the afterbirth, also to leave it intact. The husband did not know what to do, but left it altogether with his wife. I wanted consultation, but it was thirty miles to the next physician. The patient asked me to "deliver the afterbirth and let follow what would," saying that once before she had an afterbirth attached and the doctor that attended her left it to rot away, which took six weeks, and she did not want to undergo such suffering any more.

I had a kettle of warm water prepared, with alum added to make it very astringent, then a basin of cold water so if hemorrhage should set in I could battle by alternate injections of hot and cold water. I also made ready to tampon with cloths saturated with the alum water. With my right hand I manipulated the fundus of womb, and at same time I pulled on cord with a force of three or four pounds. The placenta came away as easily as any I have ever delivered. The womb contracted to a normal size, at such time. I made pressure over womb for one or two minutes, and the lady said she felt very well, much better than she expected.

I fixed a little medicine for after-pain. About the time it was to be taken she said she was wasting. I examined her and found she was flooding. I immediately gave her an injection of the previously-prepared alum water, and applied cold cloths externally. This caused the mouth of womb to close, and she said she felt better again.

I examined her and found no hemorrhage that could be seen. I waited a few minutes watching the patient, and pretty soon I saw her gape. I asked if she had any unpleasant feeling, to which she replied, "no." Another gape followed. I made another examination and found no visible hemorrhage. I then manipulated over the womb, found it rapidly enlarging. I put on a bandage and used all means at hand to cause a contraction, but utterly failed, the patient gaping every two or three minutes until she was relieved by death from internal hemorrhage, which never made its appearance externally.—*The Medical Progress, Vol. 14, No. 153, pp. 621 and 622.*

Comment is unnecessary; but we commend the article to the careful consideration of any doctor who could entertain a doubt as to what he would do under similar circumstances. —*American Practitioner and News.*

---

A case of excessive venery in a boy of thirteen years is reported to the *Louisville Medical Monthly* by Dr. Leon Solomon, assistant in pediatrics in the Kentucky School of Medicine. The boy said that for more than a year he had been regularly indulging in sexual exercise, first with one, and later with four young girls, ranging in years from twelve to sixteen. They would come to him singly, and in pairs, and beg for gratification, to which demand he acceded, when he was able, and as often as he was able. How one of his tender years could accomplish so severe a task is the wonder, yet it seems, according to the doctor's account, he was ordinarily equal to the demands of the occasion.—*Medical Age.*

